# Exploration of symptom dimensions and duration of untreated psychosis within a staging model of schizophrenia spectrum disorders

**DOI:** 10.1111/eip.13006

**Published:** 2020-06-17

**Authors:** Steven Berendsen, Henricus L. Van, Jasper W. van der Paardt, Olav R. de Peuter, Marion van Bruggen, Hans Nusselder, Margje Jalink, Jaap Peen, Jack J. M. Dekker, Lieuwe de Haan

**Affiliations:** ^1^ Department of Research Arkin Mental Health Care Amsterdam The Netherlands; ^2^ Department of Clinical Psychology University Medical Center Amsterdam, location Free University Amsterdam The Netherlands; ^3^ Department of Psychiatry University Medical Center Amsterdam, location Academic Medical Center Amsterdam The Netherlands

**Keywords:** clinical validity, duration of untreated psychosis, schizophrenia spectrum disorders, staging, symptom dimensions

## Abstract

**Aim:**

Clinical staging of schizophrenia entails a new method that identifies clusters of symptoms and variation in level of remission, with the goal to create a framework for early intervention. Additionally, duration of untreated psychosis (DUP) may influence symptom severity in the first episode of psychosis (FEP) and could necessitate refining of the staging model. However, consistent evidence concerning variation in symptom severity and DUP between stages is missing. Therefore, we evaluated the clinical validity of the staging model by investigating differences in symptom severity across stages in schizophrenia spectrum disorders. Second, we assessed if a prolonged DUP is associated with higher symptom severity in FEP.

**Methods:**

We performed a cross‐sectional study of 291 acutely admitted patients with a schizophrenia spectrum disorder. Patients were assigned to clinical stages following the definition of McGorry. Symptom severity was evaluated with the new DSM‐5 Clinician‐Rated Dimensions of Psychosis Symptom Severity (CRDPSS). In FEP, we determined the DUP.

**Results:**

Significantly higher severity scores of CRDPSS items hallucinations (*H* = 14.34, df = 4, *P*‐value = .006), negative symptoms (*H* = 19.678, df = 4, *P*‐value = .001) and impaired cognition (*H* = 26.294, df = 4, *P*‐value = <.001) were found in more advanced stages of disease. Moreover, patients with FEP and a DUP longer than 1 year showed significantly more severe negative symptoms (*U* = 314 000, *P* = .015) compared to patients with a DUP shorter than 1 year.

**Conclusions:**

The present study found supporting evidence for the clinical validity of the staging model in schizophrenia spectrum disorders. In addition, we found support for refining the stage “first episode” with information concerning the DUP.

## 1 INTRODUCTION

Although numerous evidence‐based treatments for schizophrenia have been developed over the last 60 years, disability or remission rates of the disease have failed to improve substantially (Jaaskelainen et al., 2013; James, 2018). It is becoming more clear that these persistent shortcomings are partly related to the heterogeneous nature of psychosis, considering that psychosis exists of different patterns of symptom clusters, wide variety of risk factors and a highly variable course (Kahn et al., 2015). Developing methods that map the variation of the disease could provide an opportunity for a more precise diagnosis (Kahn, 2018; McGorry, Nelson, Goldstone, & Yung, 2010). The goal is to move toward an enhanced understanding of the prognosis of schizophrenia spectrum disorders in an individual patient, which may enable us to select interventions more accurately.

To achieve more precise diagnosis, clinical staging models have been developed for schizophrenia spectrum disorders (McGorry, 2007; McGorry et al., 2010). In particular, staging models aim to refine the diagnosis of schizophrenia and other psychotic disorders by distinguishing different stages of disease in terms of type or severity of psychopathology and level of remission (Hickie et al., 2013). Eventually, the ultimate goal of staging is to establish a dynamic frame work in which patients can cross back and forward to different stages depending on their current remission status, and level of functioning. This may promote stage‐dependent treatment (McGorry, 2007). In fact, staged treatment for the pre‐clinical stages already demonstrated major benefits for patients at‐risk for psychosis (McGorry & Mei, 2018).

In recent years, clinical staging of schizophrenia spectrum disorders has received increased attention in scientific literature. Three exploratory validation studies of the staging model in schizophrenia demonstrated that more advanced stages of disease have higher severity scores at symptom level and a wide variety of clinical profilers (Berendsen et al., 2018; Godin et al., 2019; Tedja, Velthorst, van Tricht, de Haan, & Group, 2017). More specifically, two of these studies suggested that advanced disease stages have higher severity scores of depression, cognitive decline and negative symptoms. On the other hand, Tedja et al. found no significant differences in total IQ between stages. In addition, the same author also found that stages assessed at baseline were not associated with changes in negative symptoms at 3 year follow‐up (Tedja et al., 2017). Therefore, further research into the validity of the staging model in schizophrenia is necessary.

Another potential target for early intervention is the duration of untreated psychosis (DUP), which has been associated with a broad range of unfavourable outcome variables in patients with a first episode of psychosis (FEP) and schizophrenia (Penttila, Jaaskelainen, Hirvonen, Isohanni, & Miettunen, 2014). In more detail, a prolonged DUP of at least 9 months to 1 year is associated with significantly more severe negative and positive symptoms at baseline and short‐term (<2 years) follow‐up (Boonstra et al., 2012; Marshall et al., 2005; Perkins, Gu, Boteva, & Lieberman, 2005). For this reason, an earlier study suggested to split stage 2 (FEP) dependent on the DUP, since the authors noticed that patients with a long DUP presented with a worse psychiatric condition in terms of symptom severity and therapy‐resistance (Berendsen et al., 2018). To the best of our knowledge no former study investigated the validity of splitting stage 2 dependent on the DUP.

In summary, we both need more robust evidence concerning the clinical validity of the staging model of schizophrenia, and exploration of the validity of refining the first episode stage. Therefore, our primary aim is to examine differences in severity for dimensional symptoms of psychosis between stages. Our secondary aim is to examine whether DUP in FEP is associated with symptom severity at admission.

## 2 METHODS

### 2.1 Study design

Consecutively admitted patients fulfilling inclusion criteria participated in a cross‐sectional study at the acute ward a psychiatric hospital in Amsterdam, the Netherlands, from 1 December 2017 to 1 April 2019. Our hospital represents a catchment area in Amsterdam, reasons for admission were mainly because of danger arising from a psychiatric disease. Five psychiatrists and 10 residents are working in the hospital and it contains 100 clinical beds. Inclusion criteria were (a) being >18 and <65 years of age, (b) fulfilling DSM‐5 criteria for a schizophrenia spectrum disorder. The sample included 291 patients with a schizophrenia spectrum disorder.

### 2.2 Procedure

Residents presented their assessment of clinical staging, the Clinician‐Rated Dimensions of Psychosis Symptom Severity (CRDPSS) and DUP during the first week of admission at the staff meeting in the presence of the psychiatrists who had seen the patient as well. A comprehensive review of patients history and psychiatric examination was discussed. The final diagnosis, clinical staging, CRDPSS and DUP were assessed during this staff meeting. All residents were trained in assessment of the clinical stages, after training inter‐rater reliability of clinical stages of 100 included patients was sufficient (intraclass correlation coefficient = 0.757 with 95% confidence interval: 0.658‐0.829). For agreement in clinical staging we chose the intraclass correlation coefficient (ICC, 2‐way, mixed‐effects model with absolute agreement) (Koo & Li, 2016).

### 2.3 Assessments

We used criteria proposed by McGorry et al. on stage assignment to appoint patients to one of the following four stages: FEP (stage 2); incomplete remission of the first episode (stage 3A); recurrent psychosis after symptomatic recovery (stage 3B); multiple relapses, incomplete remission (stage 3C); chronic, severe persisting or unremitting illness (stage 4). We interpreted stage 3B as having recurrent psychosis with one or more episodes with symptomatic remission in between episodes. There is sufficient evidence that symptomatic remission after multiple episodes is achievable (Albus, 2012; Wiersma, Nienhuis, Slooff, & Giel, 1998). Patients with the first two clinical stages, that is, increased risk of psychosis or mild nonpsychotic symptoms are not admitted at the hospital and thus not represented in the sample.

For determination of symptom severity, we used the CRDPSS. The CRDPSS has eight items that are rated from zero to four on a Likert‐type scale, zero means not present, one is equivocal, two is present and mild, three is present and moderate and four is present and severe. We used the Dutch version of the CRDPSS, this version has been translated from the original English version by the Dutch Psychiatric Association (APA, 2014).

The DUP was based on a clinical interview with the patient, their first representatives and general practitioner. After that, we distinguished stage 2A: less than 1 year and a stage 2B: more than 1 year of the DUP. The cut‐off point of a year is in line with research demonstrating that patients with a DUP longer than 12 months have more severe symptomatology (Boonstra et al., 2012; Marshall et al., 2005; Perkins et al., 2005).

To determine the stage of disease and CRDPSS the medical doctor and psychiatrist retrieved relevant information by reviewing the medical record, an interview with the patient and relatives, ambulatory psychiatrists and general practitioner. The determination of the stage of illness was dependent on remission status in the past, number of episodes and earlier antipsychotic treatment response, and not on severity of current symptoms. With the CRDPSS, we assessed the maximum symptom severity of the first 7 days of admission, this was done by conducting psychiatric examination by the psychiatrist with the medical doctor. Consequently, stage assignment and symptom severity by the CRDPSS were depended on different data. Moreover, the hypothesis of a potential association between stage of illness and symptom severity was not known by both the medical doctor and the psychiatrists who performed the assessment. Nevertheless, we should acknowledge the limitation that assessment of both measures was not completely independent.

### 2.4 Statistical analysis

Differences in clinical and demographic characteristics were analysed by using the *X* ^2^ test, Mann‐Whitney *U* test or analysis of variance. Differences in severity scores of CRDPSS items between stages were analysed using Kruskall‐Wallis. For differences between clinical stages in symptom dimensions we used Mann‐Whitney *U* tests with a Bonferroni‐correction (0.05/5 = 0.01). Differences between stage 2A and 2B were analysed by Mann‐Whitney *U* tests with a Bonferroni‐correction (0.05/2 = 0.025). We used Kruskall‐Wallis rank analysis of covariance (Quade's test) to assess the influence of age and gender on the results (IBM, n.d.). Statistical analyses were performed with Statistical Package for the Social Sciences (SPSS) version 22.

### 2.5 Ethic approval

The Dutch Central Medical Ethical Committee has ruled that Dutch Law regarding research with humans does not apply to the collection of anonymized information and, consequently, analysing anonymized data for the present study does not require additional informed consent from participants.

## 3 RESULTS

### 3.1 Demographic and clinical characteristics

The total sample contained 291 inpatients of whom we have the clinical stage and CRDPSS rating. During the study period a total of 600 patients were admitted in our hospital. Main reason for exclusion was patients not diagnosed with a DSM‐5 diagnosis of a schizophrenia spectrum disorder (N = 206) or a missing assessment of the clinical stages and CRDPSS rating (N = 103). Patients with missing assessments of the stages or CRDPSS did not significantly differ in terms of age, gender, diagnosis of schizophrenia or global assessment of functioning compared to included patients.

In Table 1, demographic and clinical characteristics are shown. Kolmogorov‐Smirnov tests indicated that severity of CRDPSS items were not normally distributed. Therefore, we used non‐parametric tests to assess differences between clinical stages. We included 62 patients in stage 2, 9 patients in stage 3A, 127 patients in stage 3B, 75 patients in stage 3C and 18 patients in stage 4. Significant differences in age (*F* = 15.09, *P* < .001) and percentage diagnosis of schizophrenia (*X* ^2^ = 79.68, *P* < .001) between stages were found. Although there appears to be a trend (*P* value = .06) toward lower scores of the Global Assessment of Functioning (GAF) in higher disease stages, no significant differences were found. Further, no significant differences in gender were found. Specific DSM‐5 diagnosis of the sample was: schizophrenia, unspecified schizophrenia spectrum disorder, schizoaffective disorder, otherwise specified schizophrenia spectrum disorder, psychotic disorder induced by substances, brief psychotic disorder and delusional disorder.

**TABLE 1:** Clinical and demographic characteristics (N total = 291)

	Stage 2 (N = 62)	Stage 3A (N = 9)	Stage 3B (N = 127)	Stage 3C (N = 75)	Stage 4 (N = 18)	Between groups	df	*P*‐value
Age (SD)	31.20 (9.03)	33.49 (10.16)	38.15 (12.28)	42.60 (11.99)	51.23 (7.67)	*F* = 15.09	4	<.001
% Female	32%	22%	39.7%	34%	29%	*X* ^2^ = 2.31		.680
% Diagnosis schizophrenia	10%	33%	54.0%	78%	89%	*X* ^2^ = 79.68		<.001
GAF (SD)	35.38 (7.91)	41.88 (7.99)	33.97 (10.36)	32.63 (6.74)	26.67 (5.77)	*F* = 2.65	4	.060
CRDPSS items								
Mania (SD)	1.25 (1.53)	0.33 (1.00)	1.52 (1.66)	1.38 (1.65)	1.06 (1.56)	*H* = 6.411	4	.171
Depression (SD)	0.38 (0.83)	0.33 (0.50)	0.66 (1.21)	0.7 (1.21)	0.18 (0.39)	*H* = 4.888	4	.299
Hallucinations (SD)	1.75 (1.56)	1.44 (1.74)	1.54 (1.68)	2.17 (1.72)	2.71 (1.61)	*H* = 14.347	4	**.006**
Delusions (SD)	3.38 (1.36)	3.00 (1.58)	3.10 (1.30)	3.21 (1.32)	3.76 (0.75)	*H* = 5.523	4	.238
Abnormal psychomotor functioning (SD)	0.78 (1.35)	0.78 (1.56)	0.74 (1.29)	0.87 (1.38)	0.59 (1.18)	*H* = 0.668	4	.955
Disorganized speech (SD)	2.05 (2.16)	0.67 (1.12)	1.40 (1.52)	1.82 (1.91)	2.18 (1.78)	*H* = 8.698	4	.069
Negative symptoms (SD)	1.66 (1.72)	2.00 (1.94)	1.21 (1.55)	2.07 (1.60)	2.41 (1.58)	*H* = 19.678	4	**.001**
Impaired cognition (SD)	1.30 (2.17)	1.00 (1.23)	1.02 (1.64)	1.61 (1.58)	3.29 (2.09)	*H* = 26.294	4	<.001

*Note*: Bonferonni corrected *p*‐value <0.01.

### 3.2 CRDPSS items across all stages

In Table 1, the individual CRDPSS items across clinical stages are shown. Significantly higher severity scores in the more advanced clinical stages are found for hallucinations (*H* = 14.34, df = 4, *P*‐value = .006), negative symptoms (*H* = 19.678, df = 4, *P*‐value = .001) and impaired cognition (*H* = 26.294, df = 4, *P*‐value = <.001).

**FIGURE 1:**
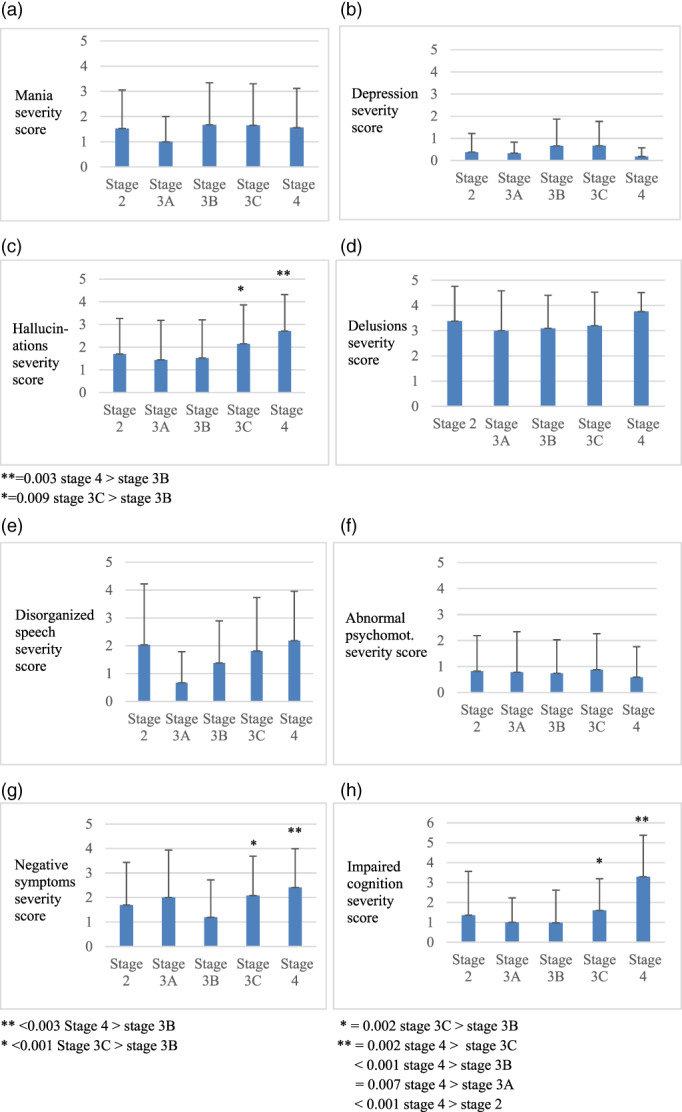
Post hoc analysis of Clinician‐Rated Dimensions of Psychosis Symptom Severity items across clinical stages

As shown in Figure 1C, higher severity hallucinations at stage 4 and 3C compared to stage 3B are found. Cognition is more impaired in stage 4 compared to stage 3C, 3B, 3A and 2. Additionally, cognitive performance in stage 3C is more affected compared to stage 3B. Further, negative symptoms demonstrated significantly higher index score in stage 4 and 3C compared to stage 3B. Considering the low number of included patients in stage 3A, we performed a sensitivity analysis by excluding this particular stage from the dataset and repeating the analysis, results did not change substantially. We repeated the Kruskall‐Wallis test with adjustments for gender and age, differences between symptom dimensions remained significant.

### 3.3 CRPDSS scores of stage 2A and 2B

As shown in Table 2, we divided stage 2 into stage 2A (N = 38) with a DUP < 1 year, and stage 2B (N = 24) with a DUP > 1 year. Of all symptom categories, only negative symptoms were significantly more severe in stage 2B compared to stage 2A (*U* = 314 000, *P* = .015).

**TABLE 2:** Clinician‐Rated Dimensions of Psychosis Symptom Severity items across stages 2A and 2B

CRDPSS items	Stage 2A (N = 38)	Stage 2B (N = 24)	Between‐groups	*P*‐value
Mania (SD)	1.43 (1.73)	0.83 (1.09)	*U* = 396 000	.221
Depression (SD)	0.22 (0.58)	0.63 (1.10)	*U* = 406 500	.173
Hallucinations (SD)	1.89 (1.60)	1.42 (1.50)	*U* = 413 000	.463
Delusions (SD)	3.30 (1.58)	3.50 (1.02)	*U* = 427 500	.432
Abnormal psychomotor functioning (SD)	0.73 (1.41)	0.96 (1.33)	*U* = 406 000	.32
Disorganized speech (SD)	2.38 (2.49)	1.50 (1.53)	*U* = 415 000	.382
Negative symptoms (SD)	1.30 (1.60)	2.29 (1.83)	*U* = 314 000	**.015**
Impaired cognition (SD)	1.19 (1.94)	1.63 (2.57)	*U* = 438 500	.569

*Note*: Bonferonni corrected *p*‐value <0.025.

## 4 DISCUSSION

The present study aimed to (a) explore the clinical validity of staging by measuring variation in symptom severity across clinical stages of patients with a schizophrenia spectrum disorder and (b) explore the validity of subdividing the first episode stage according d. Our findings showed substantially higher severity scores of impaired cognition, negative symptoms and hallucinations in the more chronic stages of schizophrenia spectrum disorders. In addition, we found substantially higher severity scores of negative symptoms in stage 2B (DUP ≥ 1 year) compared to stage 2A (DUP < 1 year) but not for the other items. Taken together, by demonstrating substantial differences in symptom profile between stages our findings support the clinical validity of the staging model. Second, we found significantly more negative symptoms in stage 2B, therefore dividing stage 2 by a short and long duration may be useful for clinical practice and prognosis.

Earlier reports regarding variation in symptom severity between clinical stages provided variable results. Two studies reported higher index severity scores of negative symptoms and cognitive decline in stage 4 compared to earlier stages (Berendsen et al., 2018; Godin et al., 2019). On the other hand, Tedja and colleagues found that baseline stages were not associated with negative symptoms at follow‐up and cognition was primarily not affected (Tedja et al., 2017). We propose two important reasons for the observed differences between studies. First, the study of Godin et al. and Tedja et al. were mainly based on a sample of outpatients with schizophrenia spectrum disorders. In contrast, our study population are acutely hospitalized patients with schizophrenia spectrum disorders and were probably more severely affected. Second, the results of Tedja and colleagues may be compromised because the authors determined the stages retrospectively and were only able to include 60% of their entire sample. This was mainly caused by lacking information concerning the level of functioning and symptomatology in the study‐cohort. Therefore, their findings could have been less generalizable.

Our findings suggested that stage 3C and 4 were both characterized by more severe negative symptoms and strong cognitive decline. Both stages represent substantial clinical challenges as currently there is no considerable evidence of an effective treatment for primary negative or cognitive symptoms (Veerman, Schulte, & de Haan, 2017). As stage 4 is defined by unremitting and chronic symptoms, our findings of increased therapy‐resistant negative and cognitive symptoms, compared to other stages, support the validity of this stage. From these findings, we advise clinicians to take into account the severe cognitive deficits that may occur in stage 4 schizophrenia, especially when evaluating for instance the feasibility for the patient of promoting work or accomplishing adequate self‐care.

With regard to hallucinations, previous studies did not find higher severity scores of hallucinations in stage 4 compared to earlier stages. On the other hand, a review investigating hallucinations in therapy‐resistant schizophrenia stated that hallucinations form a major component of the symptoms of these patients (Faden & Citrome, 2019). In line with this study, our results indicated that hallucinations were more severe in stage 4 and 3C compared to earlier stages. Perhaps, these remarkable differences in severity of hallucinations might be explained by differences in included patients: our population of acute inpatients is more severely affected by the disorder, whereas earlier studies contain predominantly outpatients with schizophrenia spectrum disorders.

Our findings indicate that distinguishing the duration of the untreated psychosis in less or more than 1 year is clinically validated by more severe negative symptoms in stage 2B. Additionally, the catchment area of Amsterdam is covered by an early detection program for patients with FEP. Therefore, a prolonged DUP could indicate higher levels of negative symptoms such as social withdraw and loss of initiative, or reluctance to seek care due to lack of disease insight among included patients. These findings are in line with meta‐analyses concerning the relationship between DUP and negative symptoms in FEP (Boonstra et al., 2012; Penttila et al., 2014). In addition, longitudinal studies demonstrated that negative symptoms at baseline are associated with important outcome parameters such as illness severity and global functioning at 12.5 year follow‐up (Moller, Bottlender, Wegner, Wittmann, & Strauss, 2000).

On the other hand, we found no significant differences in severity of other dimensions of psychosis between stage 2A and 2B. In fact, two earlier meta‐analysis demonstrated conflicting results concerning the association of positive symptoms with a longer DUP (Marshall et al., 2005; Perkins et al., 2005).

Given the above, we conclude that dividing stage 2 significantly reduces heterogeneity of symptoms in patients with FEP and could provide clinicians with important information regarding current severity of negative symptoms. Therefore, dividing stage 2 is an useful step toward personalizing diagnosis for patients with FEP.

The main strength of our study is the large number of consecutively included patients from an acute ward, which reduces selection bias. In addition, this is the first study to explore differences in severity of symptom dimensions assessed by CRDPSS between clinical stages of inpatients with schizophrenia spectrum disorders. Another strength is the training procedure of all medical doctors which resulted in sufficient inter‐rater reliability scores of clinical stages.

Nevertheless, we should acknowledge several limitations of our study. First, CRDPSS measurements and DUP assessment were a consensus rating and we did not independently assess the inter‐rater reliability of the assessors. Second, of 103 patients the CRDPSS rating or stages were missing, which was mainly because of organizational reasons such as incidental administrative flaws or absence of the treating medical doctors at the general staff meeting. We consider the sample of included patients as representative since they did not differ in terms of age, gender, diagnosis or global assessment of functioning compared to patients of whom CRDPSS or staging assessment was missing. Third, only six patients of stage 2 were diagnosed with schizophrenia, which is remarkable considering the prolonged DUP among 24 patients. The psychiatric diagnosis was based upon clinical judgement of experienced clinical psychiatrists during the first week of admission. However, we used no structured interview to confirm the diagnosis, which could have led to an underestimation of the diagnosis of schizophrenia in stage 2. Fourth, we had no additional data concerning antipsychotic medication use at admission, although most patients did not use antipsychotic treatment at admission. Therefore, we were not able to estimate a potential influence of antipsychotic treatment on symptom severity.

In conclusion, our finding supports clinical validity of the staging model in schizophrenia spectrum disorders by showing important differences between stages in symptom severity. Moreover, dividing stage 2 dependent on the DUP provides clinically relevant differences for severity of negative symptoms. Accordingly, when available, stage‐dependent treatment should focus on specific symptoms of different stages. Future research should further explore the predictive validity of staging. This type of research may further underline the large potential of staging for personalizing diagnosis, and eventually provide a framework for precision psychiatry and stage‐dependent treatment for patients with schizophrenia spectrum disorders.

## AUTHOR CONTRIBUTIONS

Steven Berendsen, Jasper W. van der Paardt, Henricus L. Van and Lieuwe de Haan contributed to the study design and proposal, literature search, analysis and interpretation. Jasper W. van der Paardt, Olav R. de Peuter, Marion van Bruggen, Hans Nusselder and Margje Jalink collected the data. Steven Berendsen drafted the manuscript and all other authors provided critical revisions. Henricus L. Van, Jaap Peen, Jack J. M. Dekker and Lieuwe de Haan supervised statistical analysis, study design and writing of the manuscript. All authors contributed to and have approved the final manuscript.

## Data Availability

The data that support the findings of this study are available on request from the corresponding author. The data are not publicly available due to privacy and ethical restrictions.
